# Relation of severe COVID-19 to polypharmacy and prescribing of psychotropic drugs: the REACT-SCOT case-control study

**DOI:** 10.1186/s12916-021-01907-8

**Published:** 2021-02-22

**Authors:** Paul M. McKeigue, Sharon Kennedy, Amanda Weir, Jen Bishop, Stuart J. McGurnaghan, David McAllister, Chris Robertson, Rachael Wood, Nazir Lone, Janet Murray, Thomas M. Caparrotta, Alison Smith-Palmer, David Goldberg, Jim McMenamin, Bruce Guthrie, Sharon Hutchinson, Helen M. Colhoun, on behalf of Public Health Scotland COVID-19 Health Protection Study Group

**Affiliations:** 1 Usher Institute, College of Medicine and Veterinary Medicine, University of Edinburgh, Teviot Place, Edinburgh, EH8 9AG Scotland; 2 Public Health Scotland, Meridian Court, 5 Cadogan Street, Glasgow, G2 6QE Scotland; 3 NHS Information Services Division (Public Health Scotland), Gyle Square, 1 South Gyle Crescent, Edinburgh, EH12 9EB Scotland; 4 Institute of Genetics and Molecular Medicine, College of Medicine and Veterinary Medicine, University of Edinburgh, Western General Hospital Campus, Crewe Road, Edinburgh, EH4 2XUC Scotland; 5 Institute of Health and Wellbeing, University of Glasgow, 1 Lilybank Gardens, Glasgow, G12 8RZ Scotland; 6 Department of Mathematics and Statistics, University of Strathclyde, 16 Richmond Street, Glasgow, G1 1XQ Scotland; 7 School of Health and Life Sciences, Glasgow Caledonian University, Glasgow, Scotland

**Keywords:** COVID-19, Pharmacoepidemiology, Antipsychotic agents, Opioids, Gabapentinoids, Proton pump inhibitors, Polypharmacy, Overprescribing

## Abstract

**Background:**

The objective of this study was to investigate the relation of severe COVID-19 to prior drug prescribing.

**Methods:**

Severe cases were defined by entry to critical care or fatal outcome. For this matched case-control study (REACT-SCOT), all 4251 cases of severe COVID-19 in Scotland since the start of the epidemic were matched for age, sex and primary care practice to 36,738 controls from the population register. Records were linked to hospital discharges since June 2015 and dispensed prescriptions issued in primary care during the last 240 days.

**Results:**

Severe COVID-19 was strongly associated with the number of non-cardiovascular drug classes dispensed. This association was strongest in those not resident in a care home, in whom the rate ratio (95% CI) associated with dispensing of 12 or more drug classes versus none was 10.8 (8.8, 13.3), and in those without any of the conditions designated as conferring increased risk of COVID-19. Of 17 drug classes postulated at the start of the epidemic to be “medications compromising COVID”, all were associated with increased risk of severe COVID-19 and these associations were present in those without any of the designated risk conditions. The fraction of cases in the population attributable to exposure to these drug classes was 38%. The largest effect was for antipsychotic agents: rate ratio 4.18 (3.42, 5.11). Other drug classes with large effects included proton pump inhibitors (rate ratio 2.20 (1.72, 2.83) for = 2 defined daily doses/day), opioids (3.66 (2.68, 5.01) for = 50 mg morphine equivalent/day) and gabapentinoids. These associations persisted after adjusting for covariates and were stronger with recent than with non-recent exposure.

**Conclusions:**

Severe COVID-19 is associated with polypharmacy and with drugs that cause sedation, respiratory depression, or dyskinesia; have anticholinergic effects; or affect the gastrointestinal system. These associations are not easily explained by co-morbidity. Measures to reduce the burden of mortality and morbidity from COVID-19 should include reinforcing existing guidance on reducing overprescribing of these drug classes and limiting inappropriate polypharmacy.

**Registration:**

ENCEPP number EUPAS35558

**Supplementary Information:**

The online version contains supplementary material available at (10.1186/s12916-021-01907-8).

## Background

At the start of the COVID-19 epidemic, possible effects of prior use of medications on the risk of severe disease were widely discussed. Some of this discussion focused on drugs suggested to be relevant specifically to SARS-CoV-2, such as drugs acting on the renin-angiotensin system [1]. Other discussion focused on drugs associated with increased risk of community-acquired pneumonia. A review published on 2 April 2020 listed 17 widely prescribed drug classes associated with increased pneumonia risk and therefore of concern as “medications compromising COVID” [2]. Excluding immunosuppressive drugs that are criteria for shielding, this list comprised proton pump inhibitors, gastrointestinal antispasmodics, H1 antihistamines, hypnotics, sedatives, antipsychotic drugs, antidepressants, drugs used in nausea and vertigo, opioid analgesics, gabapentinoids, anti-epileptic drugs, antimuscarinic drugs used in parkinsonism, urinary antispasmodics and non-steroidal anti-inflammatory drugs.

In initial analysis of a matched case-control study of risk factors for severe COVID-19 in Scotland (REACT-SCOT), we reported a strong association of severe COVID-19 with dispensing of at least one prescription in the past year [3]. The univariate rate ratios associated with at least one prescription varied from 3.8 in those aged under 60 years to 2.3 in those aged 75 years and over. This association persisted after adjusting for care home residence, hospital admission in the last 5 years and diagnoses of conditions designated by public health agencies as conferring vulnerability to COVID-19 (hereafter listed conditions). The objective of this study was to investigate what drug classes underlie this association and whether any causal effects underlie these associations of severe COVID-19 with dispensing of prescribed drugs.

## Methods

The design of the REACT-SCOT case-control study has been described in detail elsewhere [3]. A matched case-control design was chosen to keep computational requirements within the limits of the resources available. Record linkage studies using NHS data in Scotland are governed by the Public Benefit and Privacy Panel for Health and Social Care which includes patient and public representatives. All individuals testing positive for nucleic acid for SARS-CoV-2 in Scotland were ascertained through the Electronic Communication of Surveillance in Scotland (ECOSS) database. Admissions to critical care were obtained from the Scottish Intensive Care Society and Audit Group (SICSAG) database that captures admission to all critical care (intensive care or high dependency) units. Death registrations were obtained from linkage to the National Register of Scotland. Severe or fatal COVID-19 was defined as (1) a positive nucleic acid test followed by entry to critical care or death within 28 days or (2) a death certificate with COVID-19 as underlying cause. This definition of severe COVID-19 used for this study ensures that case ascertainment is not affected by triage of those assessed as unlikely (on the basis of age and underlying conditions) to benefit from critical care.

For each case, the Community Health Index database was used to select up to ten controls matched for sex, 1-year age band, and registered with the same primary care practice, who were alive and had not yet tested positive on the date that the case first tested positive. With this incidence density sampling design, the conditional odds ratios are interpretable as rate ratios for severe COVID-19 associated with exposure in the population at risk (defined as all those who have not yet tested positive).

For fatal cases who had not tested positive, the incident date was assigned as 14 days before death. For this analysis based on ascertainment of positive test results up to 6 June 2020, entry to critical care up to 14 June 2020 and deaths up to 12 June 2020, there were 4251 cases and 36,738 controls.

### Morbidity and drug prescribing

For all cases and controls, ICD-10 diagnostic codes were extracted from the last 5 years of hospital discharge records in the Scottish Morbidity Record (SMR01), excluding records of discharges less than 25 days before testing positive for SARS-CoV-2, and from the national cancer registry. Diagnoses of diabetes were extracted from linkage to the national diabetes register. British National Formulary (BNF) drug codes for dispensed prescriptions issued in primary care were extracted from the Scottish Prescribing Information System [4]. A cutoff date of 15 days before the incident date (date of testing positive for SARS-CoV-2, or 14 days before death for fatal cases without a positive test) was set, and prescriptions dispensed in a 240-day interval before this cutoff date were included. For this analysis prescription codes from BNF chapters 14 and above, comprising dressings, appliances, vaccines, anaesthesia and other preparations were grouped as “Other”.

We began by testing for association of severe COVID-19 with the number of drug classes (BNF subparagraph codes) for which at least one prescription had been dispensed during the period of observation. In accordance with earlier suggestions that prescribing of multiple cardiovascular drugs, which is supported by evidence-based guidelines, should be considered separately from putatively inappropriate polypharmacy [5], we partitioned the number of drug classes dispensed into cardiovascular and other drugs. We then undertook a systematic study of associations will all drug classes and subsequently a test of the drug classes postulated to be “medications compromising COVID” [2].

For proton pump inhibitors and opioids, it was possible to use equivalent doses to calculate a total dose for all drugs in the class. Defined daily doses (DDDs) of each proton pump inhibitor were obtained from the DDD/ATCIndex of the WHO Collaborating Centre for Drug Statistics Methodology. Morphine milligram equivalents (MMEs) for opioids were obtained from the website of the FacultyofPainMedicine of the Royal College of Anaesthestists. Average daily doses of proton pump inhibitors (as DDDs) and opioids (as MME) were calculated for each individual as the sum of (conversion factor × strength × quantity dispensed) divided by observation period. The dose of opioid was calculated as the sum over opioid-containing items in subparagraph 0407010 (nonopioid and compound preparations) and 0407020 (opioid analgesics).

As described previously, we derived indicator variables for a list of conditions that have been designated as risk conditions for COVID-19 by public health agencies [6]: diabetes, heart disease, asthma or chronic obstructive airway disease, chronic kidney disease, disabling neurological disease, liver disease and immunodeficiency or immunosuppression. ICD-10 diagnostic and BNF drug codes used to derive these conditions are available with the ENCEPPregistration . Socioeconomic status was encoded as the quintile of the postcode-based Scottish Index of Multiple Deprivation (SIMD).

### Statistical analysis

Rate ratios for severe COVID-19 (hereafter COVID-19) were estimated from conditional logistic regression models, implemented as Cox regression in the R function survival::clogit. To estimate the population attributable fraction—the fraction of cases in the population that are attributable to the exposure if the association between exposure and disease is causal—for a group of drugs, we fitted a model including these drug classes together with other covariates and computed the difference *η* between the linear predictors from this model with drug exposures set to their observed values and the linear predictors from the same model with drug exposures set to zero. For a rare disease, *e*^*η*^/(1+*e*^*η*^) approximates the population attributable risk fraction [7].

As we had previously shown that care home residence is associated with a rate ratio of more than 20 for severe COVID-19, and the profile of prescribing in care home residents is likely to be different from that in the general population, most analyses of the relation of severe COVID-19 to drug exposure were restricted to those not resident in a care home.

To control for confounding by co-morbidity, most analyses of association of severe COVID-19 used restriction to those without any of the designated risk conditions, rather than adjustment for covariates. The rationale for this was that electronic health records of drug prescribing and hospital admissions do not provide information on the severity of co-morbid conditions, but do contain enough information to assign the presence or absence of each of the designated risk conditions. For some specific drug classes, we were able to define a prespecified list of relevant covariates.

To distinguish between causality and confounding as alternative explanations for associations of severe COVID-19 with drug exposure, we used several further approaches:

- Testing for consistency of association with drugs that have a similar mode of action across different indication groups
- Testing for a dose-response relationship and stratifying by age group
- Comparison between recent and less recent time windows of dispensing. Because of limits on the computational resources available for this study, the extract of prescription data was limited to prescriptions encashed from 1 July 2019. An interval of 240 days, split into two equal windows of 120 days, was chosen so that there was no left-censoring. The classic case-crossover design, which compares in cases only the frequencies of exposure in recent and nonrecent time windows, can be viewed as a matched-pairs case-control study in which the case and control are the same person in recent and nonrecent time windows [8]. The conditional odds ratio can thus be estimated as the ratio of number of cases with recent exposure only to number of cases with nonrecent exposure only. The analysis that we report is a refinement of this discordant-pairs ratio estimate that takes advantage of the availability of a matched control group. We contrast not the numbers of cases with recent exposure only and nonrecent exposure only, but the conditional odds of case status between those with recent exposure only and those with nonrecent exposure only. This controls for any difference in the population frequencies of exposure between recent and nonrecent time windows.

## Results

Table 1 compares severe non-fatal and fatal cases by test-positive and entry to critical care. Table 2 shows frequencies of risk factors in cases and controls by age group.

**Table 1:** Comparison of severe non-fatal and fatal cases, by test-positive status and entry to critical care

	Non-fatal	Fatal		
	Test-positive			No positive test
	Critical care, non-fatal (462)	Critical care, fatal (240)	No critical care, fatal (2223)	No critical care, fatal (1310)
Age [median (IQR)]	59 (50.25–65)	65.5 (58–73)	83 (76–88)	84 (76–89)
Males	305 (66%)	187 (78%)	1111 (50%)	586 (45%)
Care home	4 (1%)	1 (0%)	1051 (47%)	838 (64%)
Any prescription	412 (89%)	223 (93%)	2180 (98%)	1280 (98%)
Any admission	253 (55%)	161 (67%)	1932 (87%)	1092 (83%)
Type 1 diabetes	10 (2%)	2 (1%)	22 (1%)	8 (1%)
Type 2 diabetes	90 (19%)	61 (25%)	503 (23%)	251 (19%)
Other/unknown type	13 (3%)	6 (2%)	22 (1%)	7 (1%)
Ischaemic heart disease	45 (10%)	31 (13%)	559 (25%)	269 (21%)
Other heart disease	63 (14%)	45 (19%)	1062 (48%)	570 (44%)
Asthma or chronic airway disease	117 (25%)	65 (27%)	799 (36%)	445 (34%)
Chronic kidney disease or transplant recipient	6 (1%)	5 (2%)	62 (3%)	23 (2%)
Neurological (except epilepsy) or dementia	20 (4%)	18 (8%)	801 (36%)	542 (41%)
Liver disease	1 (0%)	4 (2%)	30 (1%)	15 (1%)
Immune deficiency or suppression	5 (1%)	4 (2%)	24 (1%)	6 (0%)

**Table 2:** Frequencies of risk factors in cases and controls, by age group

	0–39 years		40–59 years		60–74 years		75+ years	
	Controls (560)	Cases (56)	Controls (4078)	Cases (409)	Controls (8634)	Cases (871)	Controls (23,466)	Cases (2915)
Care home	0 (0%)	1 (2%)	4 (0%)	19 (5%)	90 (1%)	176 (20%)	2841 (12%)	1698 (58%)
Any prescription	308 (55%)	47 (84%)	2862 (70%)	362 (89%)	7514 (87%)	830 (95%)	22656 (97%)	2871 (98%)
Any admission	140 (25%)	26 (46%)	1454 (36%)	246 (60%)	4348 (50%)	667 (77%)	16522 (70%)	2507 (86%)
Any listed condition	63 (11%)	29 (52%)	1012 (25%)	220 (54%)	3727 (43%)	634 (73%)	14296 (61%)	2436 (84%)
Diagnosis or prescription	339 (61%)	48 (86%)	3058 (75%)	377 (92%)	7734 (90%)	850 (98%)	22885 (98%)	2900 (99%)
Type 1 diabetes	0 (0%)	2 (4%)	46 (1%)	12 (3%)	42 (0%)	8 (1%)	70 (0%)	21 (1%)
Type 2 diabetes	3 (1%)	2 (4%)	246 (6%)	72 (18%)	1305 (15%)	219 (25%)	3970 (17%)	613 (21%)
Other/unknown type	2 (0%)	4 (7%)	22 (1%)	14 (3%)	73 (1%)	8 (1%)	184 (1%)	22 (1%)
Ischaemic heart disease	2 (0%)	0 (0%)	125 (3%)	33 (8%)	948 (11%)	169 (19%)	4391 (19%)	702 (24%)
Other heart disease	6 (1%)	7 (12%)	174 (4%)	64 (16%)	1225 (14%)	263 (30%)	7190 (31%)	1411 (48%)
Asthma or chronic airway disease	48 (9%)	22 (39%)	560 (14%)	112 (27%)	1669 (19%)	326 (37%)	5305 (23%)	970 (33%)
Chronic kidney disease or transplant recipient	1 (0%)	0 (0%)	8 (0%)	15 (4%)	30 (0%)	24 (3%)	163 (1%)	57 (2%)
Neurological (except epilepsy) or dementia	3 (1%)	7 (12%)	60 (1%)	43 (11%)	319 (4%)	177 (20%)	2897 (12%)	1154 (40%)
Liver disease	1 (0%)	0 (0%)	20 (0%)	10 (2%)	52 (1%)	21 (2%)	59 (0%)	20 (1%)
Immune deficiency or suppression	2 (0%)	1 (2%)	18 (0%)	12 (3%)	47 (1%)	15 (2%)	76 (0%)	11 (0%)

### Relation of severe COVID-19 to polypharmacy

**Fig. 1:**
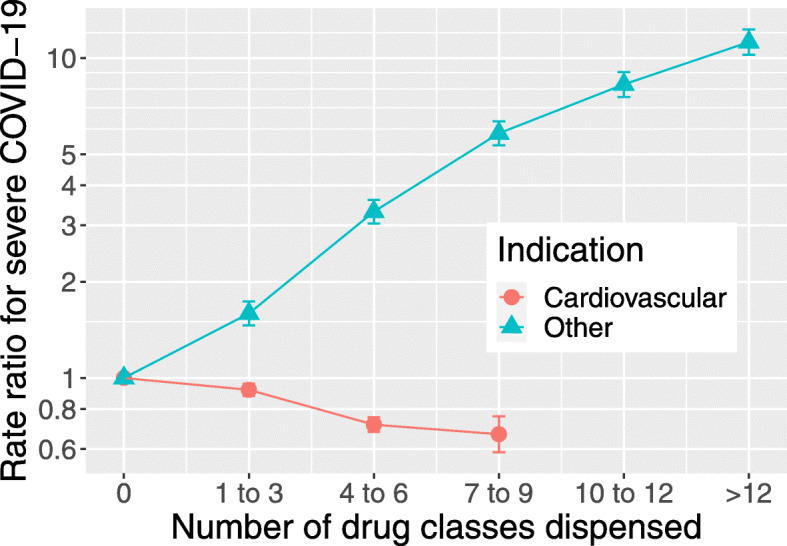
Rate ratios (with standard errors) in a conditional logistic regression of severe COVID-19 on number of cardiovascular (BNF chapter 4) and non-cardiovascular drug classes dispensed

Figure 1 shows that the rate of severe COVID-19 increased steeply with the number of non-cardiovascular drug classes and decreased with the number of cardiovascular drug classes dispensed. Table 3 shows that this association was restricted to those not resident in a care home. Among those not resident in a care home, the association of severe COVID-19 with the number of non-cardiovascular drug classes dispensed remained after stratifying by the presence or absence of at least one listed condition (Supplementary Table S1). This association was stronger in those aged less than 75 years than in those aged 75 years and over (Supplementary Table S2).

**Table 3:** Association of severe COVID-19 with number of non-cardiovascular drug classes dispensed, by care home residence

Number of drug classes	Controls (36,738)	Cases (4251)	Rate ratio (95% CI)	*p* value
**Not resident in care home (33,803 controls, 2357 cases)**				
0	4962 (15%)	167 (7%)		
1 to 3	11,036 (33%)	442 (19%)	1.56 (1.29, 1.88)	4×10^−6^
4 to 6	8492 (25%)	578 (25%)	2.97 (2.47, 3.59)	4×10^−30^
7 to 9	5083 (15%)	469 (20%)	4.45 (3.66, 5.42)	3×10^−50^
10 to 12	2511 (7%)	328 (14%)	6.6 (5.4, 8.2)	2×10^−69^
>12	1719 (5%)	373 (16%)	10.8 (8.8, 13.3)	5×10^−110^
**Care home residents (2935 controls, 1894 cases)**				
0	40 (1%)	32 (2%)		
1 to 3	261 (9%)	167 (9%)	2.01 (0.87, 4.63)	0.1
4 to 6	800 (27%)	395 (21%)	1.38 (0.62, 3.09)	0.4
7 to 9	821 (28%)	574 (30%)	1.86 (0.83, 4.17)	0.1
10 to 12	585 (20%)	425 (22%)	2.02 (0.90, 4.55)	0.09
>12	428 (15%)	301 (16%)	1.91 (0.85, 4.30)	0.1

### Associations with specific drug classes

Supplementary Table S3 shows that among controls aged over 75 years dispensed prescriptions of many drug classes were more frequent in care home residents than in those living independently. For the next step in the analysis, cases and controls were restricted to those not resident in a care home. Table 4 shows univariate associations of dispensing of at least one drug in each BNF subparagraph, filtered to show only drug classes with at least 50 exposed individuals and *p* < 0.001. The drug classes associated with severe COVID-19 include proton pump inhibitors, laxatives, multiple classes of drugs acting on the central nervous system, nutritional supplements and non-steroidal anti-inflammatory drugs.

**Table 4:** Univariate rate ratios for severe COVID-19 associated with dispensing of each BNF subparagraph code, restricted to those without a listed condition and not resident in a care home, filtered to show only drug classes with at least 50 exposed individuals and *p* < 0.001

BNF subparagraph	Controls (17,127)	Cases (637)	Rate ratio	*p* value
103050: Proton pump inhibitors	4643	228	1.84 (1.51, 2.23)	7×10^−10^
104020: Antimotility drugs	262	20	2.84 (1.60, 5.05)	4×10^−4^
105010: Aminosalicylates	148	15	3.55 (1.83, 6.89)	2×10^−4^
106040: Osmotic laxatives	1251	78	2.33 (1.71, 3.18)	1×10^−7^
401020: Anxiolytics	485	41	2.17 (1.46, 3.23)	1×10^−4^
402010: Antipsychotic drugs	165	25	3.89 (2.29, 6.62)	5×10^−7^
403010: Tricyclic and related antidepressant drugs	1037	64	1.86 (1.36, 2.54)	9×10^−5^
403040: Other antidepressant drugs	498	48	2.74 (1.89, 3.97)	1×10^−7^
407010: Non-opioid analgesics and compound preparations	4202	223	2.05 (1.68, 2.50)	1×10^−12^
407020: Opioid analgesics	1028	79	2.32 (1.72, 3.13)	3×10^−8^
408010: Control of epilepsy	746	51	2.31 (1.63, 3.28)	3×10^−6^
501012: Penicillinase-resistant penicillins	571	41	2.28 (1.53, 3.39)	5×10^−5^
501013: Broad-spectrum penicillins	1155	69	1.92 (1.42, 2.62)	3×10^−5^
603020: Use of corticosteroids	333	33	3.36 (2.11, 5.35)	3×10^−7^
901011: Oral iron	409	26	2.81 (1.66, 4.78)	1×10^−4^
901020: Drugs used in megaloblastic anaemias	1018	56	2.14 (1.51, 3.04)	2×10^−5^
904020: Enteral nutrition	99	17	5.8 (2.7, 12.2)	5×10^−6^
906026: Thiamine hydrochloride (B1)	122	15	3.11 (1.59, 6.07)	9×10^−4^
906040: Vitamin D	1730	73	1.85 (1.33, 2.58)	3×10^−4^
1001010: Non-steroidal anti-inflammatory drugs	1438	86	1.71 (1.31, 2.22)	7×10^−5^
1003020: Rubefacients, topical NSAIDS, capsaicin and poultice	1780	80	1.61 (1.21, 2.14)	1×10^−3^

Table 5 shows associations of severe COVID-19 with dispensing of each of the drug classes on the prespecified list of “medications compromising COVID” [2], restricted to those not resident in a care home. All drug classes on this list were associated with increased risk in univariate analyses (though the association with NSAIDS was stronger in Table 4 where listed conditions were excluded). This included all the drug classes listed by Laporte and Healy [2] as having anticholinergic effects likely to increase risk of pneumonia: H1 antihistamines, antidepressants, urinary antispasmodics, gastrointestinal antispasmodics, drugs for vertigo, antimuscarinic drugs used in the treatment of parkinsonism and antiepileptic drugs. In a conditional logistic regression model with care home residence as covariate, the fraction of cases of severe COVID-19 attributable to exposure to drug classes on the Laporte-Healy list was estimated as 38%.

**Table 5:** Associations of severe COVID-19 with drug classes listed by Laporte and Healy (2020), restricted to those not resident in a care home

			Univariate		Adjusted	
	Controls (33,803)	Cases (2357)	Rate ratio (95% CI)	*p* value	Rate ratio (95% CI)	*p* value
103050.Proton pump inhibitors	12,745 (38%)	1168 (50%)	1.81 (1.66, 1.98)	7×10^−39^	1.44 (1.31, 1.58)	1×10^−13^
102000.Antispasmodic and other drugs altering gut motility	1226 (4%)	132 (6%)	1.73 (1.42, 2.10)	3×10^−8^	1.07 (0.87, 1.32)	0.5
304010.Antihistamines	2556 (8%)	263 (11%)	1.61 (1.40, 1.85)	4×10^−11^	1.30 (1.13, 1.51)	4×10^−4^
401010.Hypnotics	1395 (4%)	157 (7%)	1.74 (1.45, 2.09)	2×10^−9^	1.01 (0.83, 1.23)	0.9
401020.Anxiolytics	1308 (4%)	184 (8%)	2.17 (1.83, 2.58)	3×10^−19^	1.25 (1.04, 1.51)	0.02
402010.Antipsychotic drugs	526 (2%)	151 (6%)	4.18 (3.42, 5.11)	6×10^−44^	2.80 (2.24, 3.51)	2×10^−19^
402030.Drugs used for mania and hypomania	67 (0%)	10 (0%)	2.53 (1.26, 5.10)	0.009	0.93 (0.43, 1.98)	0.8
403010.Tricyclic and related antidepressant drugs	2761 (8%)	268 (11%)	1.59 (1.38, 1.83)	1×10^−10^	1.10 (0.94, 1.27)	0.2
403030.Selective serotonin re-uptake inhibitors	2913 (9%)	300 (13%)	1.60 (1.40, 1.83)	4×10^−12^	1.18 (1.03, 1.36)	0.02
403040.Other antidepressant drugs	1561 (5%)	259 (11%)	2.71 (2.34, 3.14)	8×10^−40^	1.76 (1.50, 2.07)	6×10^−12^
406000.Drugs used in nausea and vertigo	1798 (5%)	213 (9%)	1.95 (1.67, 2.29)	1×10^−16^	1.42 (1.20, 1.68)	4×10^−5^
407020.Opioid analgesics	3371 (10%)	483 (20%)	2.60 (2.32, 2.92)	1×10^−59^	1.83 (1.61, 2.08)	1×10^−20^
Gabapentinoids	1742 (5%)	253 (11%)	2.29 (1.98, 2.65)	1×10^−28^	1.38 (1.18, 1.62)	8×10^−5^
Other drugs used for epilepsy	754 (2%)	100 (4%)	1.85 (1.48, 2.30)	5×10^−8^	1.36 (1.08, 1.71)	0.009
409020.Antimuscarinic drugs used in parkinsonism	30 (0%)	7 (0%)	2.97 (1.26, 7.03)	0.01	0.90 (0.37, 2.21)	0.8
704020.Drugs for urinary frequency enuresis and incontinence	1626 (5%)	133 (6%)	1.36 (1.12, 1.64)	0.002	1.02 (0.84, 1.24)	0.8
1001010.Non-steroidal anti-inflammatory drugs	2373 (7%)	206 (9%)	1.17 (1.00, 1.36)	0.05	0.88 (0.75, 1.03)	0.1

Multivariate rate ratios are based on a joint model with all drug classes in the table and SIMD quintile as covariates

In a multivariable regression, the strongest independent associations were with proton pump inhibitors, antihistamines, antipsychotic drugs and opioid analgesics. In both univariate and multivariable analyses, the highest rate ratio was that associated with antipsychotic drugs: univariate rate ratio (95% CI) 4.18 (3.42, 5.11).

As others have noted [2], the chemical structures and modes of action of drugs used in the treatment of nausea and vertigo overlap with those of antipsychotic drugs. Supplementary Table S4 shows the univariate associations of severe COVID-19 with specific drugs classified in these two subparagraphs of the BNF. Across both groups of indications, phenothiazines and other drugs that are dopamine antagonists were strongly associated with increased rates of severe disease. Rate ratios were elevated both for phenothiazines and for second-generation antipsychotics: aripiprazole, olanzapine, risperidone and amrisulpride.

### Time window analyses

Supplementary Table S5 shows for each drug class on the Laporte-Healy list the rate ratio associated with dispensing in the most recent 120-day time window, with dispensing only in the previous time window as reference category. Because most users of these drugs had dispensed prescriptions in both time windows, these analyses are based on relatively small numbers: the drug classes shown are restricted to those with at least 500 cases and controls exposed only in the earlier time window. For all of the drug classes shown, the rate ratio associated with recent exposure only is above 1, but only for opioid analgesics does this association reach stringent levels of statistical significance.

### Dose-response analyses

Supplementary Table S6 shows the relationship of severe COVID-19 to average daily doses of opioids (as MME) and proton pump inhibitors (as DDDs) over the 240-day observation period. For both these drug classes, there were dose-response relationships, and for proton pump inhibitors, this relationship was strongest in those aged less than 75 years. With unexposed as baseline, the univariate rate ratio associated with opioid use in this age group was 3.66 (2.68, 5.01) in those with average daily dose of more than 50 mg morphine equivalent (MME), reduced to 2.96 (2.08, 4.20) on adjusting for care home residence, SIMD quintile and any history of neoplasm. For proton pump inhibitors, the univariate rate ratio associated with average dose of 2 or more DDDs/day was 2.20 (1.72, 2.83), reduced to 1.99 (1.51, 2.62) by adjusting for care home residence, SIMD quintile, any diagnosis of ICD-10 codes K20-K31 (diseases of oesophagus, stomach and duodenum), non-steroidal anti-inflammatory drugs, anti-platelet agents and anticoagulants.

### Associations with other drug classes

Supplementary Tables S7 and S8 show associations with drug classes in BNF chapters 2 (cardiovascular) and 10 (musculoskeletal). These chapters were selected as of interest because specific hypotheses about possible effects of drugs in these chapters—ACE inhibitors [9], anticoagulants [10] and hydroxychloroquine [11]—have been proposed or discussed.

Table S7 shows associations with drugs for the cardiovascular system. Prescriptions of loop diuretics and anticoagulants were associated with elevated rate ratios for severe COVID-19 in univariate and multivariate analyses. Over all age groups combined, the univariate rate ratio associated with oral anticoagulants was reduced from 1.87 (1.65, 2.11) to 1.27 (1.11, 1.45) by adjustment for ischaemic heart disease, other heart disease and ever-use of a proton pump inhibitor. Of drug classes commonly used to treat hypertension, ACE inhibitors and angiotensin II receptor antagonists were associated with reduced risk of COVID-19.

Supplementary Table S8 shows associations with drugs for the musculoskeletal system disaggregated by generic name, as the BNF groups all disease-modifying antirheumatic drugs under a single subparagraph code. All antirheumatic drugs were associated with elevated rate ratios for COVID-19. The univariate rate ratio associated with hydroxychloroquine sulfate was adjusted for ever-use of a proton pump inhibitor reducing this to 1.82 (1.16, 2.85).

## Discussion

We have shown that severe COVID-19 is associated with polypharmacy, defined by the number of drug classes dispensed during the period of observation, in individuals without conditions designated as conferring high risk. The rate ratios of 5 to 7 associated with dispensing of more than 10 drug classes are larger than the ratio of about 2 for all-cause mortality associated with this level of polypharmacy in a systematic review [12]. Attempting to investigate associations with specific drugs with a hypothesis-free approach is difficult because many of the drug classes that are strongly associated with severe COVID-19, such as proton pump inhibitors, opioids and gabapentinoids, are indicators of overprescribing, recognized as such in the Scottish National Therapeutic Index of prescribing quality [13].To narrow the hypothesis space, we tested a pre-specified list of drugs postulated at the start of the epidemic to increase risk of severe COVID-19, based on previously described associations with pneumonia or activity on relevant pathways, especially anticholinergic agents [2]. We have shown that all the drugs originally listed are associated with increased risk, but the strongest associations are with antipsychotic drugs. The dose-response relationship of opioid use to COVID-19 is similar in magnitude to that reported for community-acquired pneumonia in a study of people receiving medical care through the Veterans Administration from 2000–2012 [14]. Although gabapentinoids are classified in the BNF under subparagraph 0408010 (“Control of epilepsy”), in Scotland, they are widely used in combination with or as substitutes for opioid analgesics.

In observational studies based on electronic health records, distinguishing between causality and confounding as possible explanations for associations of adverse outcomes with drug exposure is challenging because such associations are confounded by the indications for which the drugs were prescribed (co-morbidities). Because the severity of these co-morbid conditions cannot usually be assessed accurately using electronic health records, control of confounding based on adjustment for baseline covariates is likely to be inadequate. We have used several approaches to overcome this limitation.

First, we have examined associations between drug exposure and severe COVID-19 after restricting to those not diagnosed with designated risk conditions for COVID-19; this makes it unnecessary to adjust for the severity of these co-morbid conditions. This use of restriction as a technique to assess evidence for causality does not affect the generalizability of the association of severe COVID-19 with polypharmacy, as this association is present in those with and without risk conditions. Where exposure-outcome associations are stronger in younger than in older individuals, as for opioids and proton pump inhibitors, this favours causality over confounding by co-morbidity as these co-morbid conditions are less common at younger ages.

Second, we have used time-window analyses, in which the comparison is restricted to ever-exposed individuals and the rate ratios associated with use only in a recent time window are compared with rate ratios associated with use only in a non-recent time window. Such “self-controlled” designs, which eliminate confounding by time-invariant factors are a standard method in pharmacoepidemiology [15]. We have shown that among ever-exposed individuals the rate ratios associated with dispensing only in the most recent time window were higher than the rate ratios associated with dispensing only in an earlier time window.

Third, we have examined dose-response relationships for two drug classes—opioids and proton pump inhibitors—where it is possible to standardize dosages across different drugs in the same class. We have shown strong dose-response relationships of COVID-19 to dispensed average daily dose. Adjustment for covariates pre-specified as likely to confound these associations reduces the effect size only slightly.

Fourth, we can examine whether exposure-outcome associations are consistent for pharmacologically similar drugs that are prescribed for different indications. We have shown that the associations of severe COVID-19 with phenothiazines and other dopamine antagonists are similar whether these drugs are prescribed as antipsychotic agents or as drugs for nausea and vomiting. As these two indications are unrelated and there is no obvious prior hypothesis that they would both predispose to severe COVID-19, this result favours causality over confounding by indication. As the rate ratio of 4.2 associated with use of antipsychotic agents is larger than the rate ratios of about 2 associated with common risk conditions such as type 2 diabetes and heart disease that we have previously reported from this case-control study [3], it is unlikely that underdiagnosis of these risk conditions in users of antipsychotic agents can explain this association.

A limitation of this study is that we do not have morbidity data from primary care, which would include risk factors such as smoking and coding of presenting complaints. Another limitation is that it is not yet possible in Scotland to capture hospital prescribing data which includes biologic agents that have immunosuppressive effects. Strengths of this study are that diagnoses are based on hospital discharge records coded to ICD-10 (rather than the SNOMED-CT codes used in primary care databases), and that drug exposure is based on dispensed rather than issued prescriptions. A report from the Swedish National Public Health Agency that compared prior drug prescribing rates in fatal cases of COVID-19 and with prescribing rates in the general population showed a similar pattern of drug classes associated with fatal disease, including antipsychotics, opioids and drugs for gastroesophageal reflux but did not include any control for covariates [16].

The mechanisms postulated by Laporte and Healy for drugs to increase risk of severe COVID-19 include sedation, respiratory depression, respiratory dyskinesia and anticholinergic effects [2]. Dose-response relationships to risk of community-acquired pneumonia have been reported for opioids [17] and for antipsychotic drugs [18]. For proton pump inhibitors, association with risk of community-acquired pneumonia for recent but not long-term use has been reported in a large study [19]. However as SARS-CoV-2 is at least partly an enteric infection [20] and the ACE2 receptor is expressed in the intestine, it is plausible that proton pump inhibitors and other drugs acting on the gastrointestinal tract could increase susceptibility to severe COVID-19 even if they do not increase risk of pneumonia caused by other infectious agents. In Scotland, overprescribing is monitored and to some extent controlled through the National Prescribing Quality Initiative. It may be relevant to investigate associations of COVID-19 with drug prescribing in other countries where COVID-19 epidemics have been especially severe and overprescribing of drug classes such as proton pump inhibitors [21–23] or opioids [24] has been reported previously.

We emphasize that because of the relationship of COVID-19 to polypharmacy, associations with specific drug classes cannot be studied without taking into account how those drug classes are related to the profile of drug prescribing. Of those drug classes that are not on the Laporte-Healy list, there are strong univariate associations of severe COVID-10 with dispensing of antibiotics, laxatives and nutritional supplements. The associations with nutritional supplements are likely to be confounded by overprescribing. For ACE inhibitors and angiotensin-II receptor blockers, our results are consistent with other studies that have found no increased risk associated with these drugs [9, 25] and indeed suggest some protective effect may be possible. To explore this more fully will require access to other datasets where measurements of blood pressure and other covariates are available. The relation of prior anticoagulant use to COVID-19 is of interest because coagulopathy is a feature of severe disease [10]. Although in this study anticoagulants were associated with increased risk in univariate analysis, this association was reduced by adjusting for covariates including diagnosed heart disease and co-prescribing of proton pump inhibitors. Associations with disease-modifying antirheumatic drugs such as hydroxychloroquine are likely to be confounded by hospital-based prescribing of biologic anti-rheumatic drugs, which is not captured by record linkage in Scotland.

## Conclusion

Severe COVID-19 is strongly associated with polypharmacy in those not resident in a care home. This association is not easily explained by co-morbidity, and it is strongest in those without hospital diagnoses of conditions that confer high risk of disease. As a prediction of which drug classes would be associated with increased susceptibility to severe COVID-19, the Laporte-Healy list prepared at the start of the epidemic appears to be remarkably accurate. Many of the drug classes on this list are recognized indicators of overprescribing. The consistency of associations with drugs that have similar modes of action across different groups of indication, the dose-response and time window effects support causal explanations for at least some of these associations. We recommend that public health agencies should reinforce existing guidelines on avoidance of overprescribing of these drug classes and more generally on inappropriate polypharmacy.

## Supplementary Information

**Additional file 1** Supplementary information.

## Data Availability

Requests for access to the data may be submitted to the Public Benefit and Privacy Panel for Health and Social Care (https://www.informationgovernance.scot.nhs.uk/pbpphsc/). All source code used for derivation of variables, statistical analysis and generation of this manuscript is freely available on GitHub. The ICD-10 and BNF drug codes used to define risk conditions are available on the ENCEPP registration page (http://www.encepp.eu/encepp/openAttachment/documents.otherDocument-0/35565).
