# Supplementary material for: Relation of severe COVID-19 to polypharmacy and prescribing of psychotropic drugs: the REACT-SCOT case-control study
Source: BMC Med. 2021 Feb 22;19:51. doi: 10.1186/s12916-021-01907-8 (PMC7897516; doi:10.1186/s12916-021-01907-8)
Supplement: Supplementary file 1 — Additional file 1 Supplementary information. [file 12916_2021_1907_MOESM1_ESM.pdf]

Supplementary information

Table S1

Association of severe COVID-19 with number of non-cardiovascular drug classes dispensed, in those not resident in a care home by diagnosis of at least one listed condition.

Table S2

Association of severe COVID-19 with number of non-cardiovascular drug classes dispensed, in those not resident in a care home by age group .

Table S3

Univariate conditional odds ratios for dispensing of BNF subparagraph codes associated with care home status in controls aged 75 years, filtered to retain rows with at least 50 exposed care home residents.

Table S4

Univariate associations of severe COVID-19 with drugs in four subparagraphs of BNF chapter 4, in those not resident in a care home and not treated for cancer in last year, filtered to retain rows with at least 20 exposed individuals.

Table S5

Comparison of associations with exposure only in the most recent 120-day time window with exposure only in the previous 120-day time window (baseline category for rate ratios) for drug classes on the Laporte-Healy list with at least 500 cases and controls exposed only in the earlier time window.

Table S6

Associations of severe COVID-19 with dispensed average daily dose of opioids and proton pump inhibitors, with and without adjustment for prespecified covariates. For opioids the prespecified covariates are care home residence, SIMD quintile and any history of neoplasm. For proton pump

inhibitors the prespecified covariates are care home residence, SIMD quintile, any diagnosis of ICD-10 codes K20-K31 (diseases of esophagus, stomach and duodenum), non-steroidal anti-inflammatory drugs, anti-platelet agents and anticoagulants.

Table S7

Associations of severe COVID-19 with prescribed drugs in BNF chapter 2 by subparagraph code, restricted to those not resident in a care home and filtered to retain rows with at least 50 exposed individuals. Multivariate rate ratios are based on a joint model with all drug classes in the table as covariates.

Table S8

Associations of severe COVID-19 with prescribed drugs in BNF chapter 10 by approved name, restricted to those not resident in a care home and filtered to retain rows with at least 50 exposed individuals. Multivariate rate ratios are based on a joint model that includes all drugs in the table as covariates.

Supplementary Tables

Table S1. Association of severe COVID-19 with number of non-cardiovascular drug classes dispensed, in those not resident in a care home by diagnosis of at least one listed condition

Number of drug classes	Controls (33803)	Cases (2357)	Rate ratio (95% CI)	p-value
**No listed condition (17127 controls, 637 cases)**				
0	4461 (26%)	120 (19%)		
1 to 3	7090 (41%)	224 (35%)	1.60 (1.26, 2.04)	1×10^{-4}
4 to 6	3552 (21%)	150 (24%)	2.70 (2.04, 3.57)	4×10^{-12}
7 to 9	1419 (8%)	79 (12%)	4.25 (2.96, 6.09)	4×10^{-15}
10 to 12	456 (3%)	46 (7%)	7.7 (4.8, 12.3)	1×10^{-17}
¿12	149 (1%)	18 (3%)	7.3 (3.8, 14.0)	2×10^{-9}
**At least one listed condition (16676 controls, 1720 cases)**				
0	501 (3%)	47 (3%)		
1 to 3	3946 (24%)	218 (13%)	0.61 (0.43, 0.88)	0.009
4 to 6	4940 (30%)	428 (25%)	1.01 (0.71, 1.43)	1
7 to 9	3664 (22%)	390 (23%)	1.34 (0.94, 1.91)	0.1
10 to 12	2055 (12%)	282 (16%)	1.87 (1.30, 2.70)	8×10^{-4}
¿12	1570 (9%)	355 (21%)	2.94 (2.05, 4.24)	6×10^{-9}

Table S2. Association of severe COVID-19 with number of non-cardiovascular drug classes dispensed, by age group in those not resident in a care home

Number of drug classes	Controls (33803)	Cases (2357)	Rate ratio (95% CI)	p-value
**Age 0-74 years (13178 controls, 1140 cases)**				
0	2632 (20%)	95 (8%)		
1 to 3	3709 (28%)	210 (18%)	1.87 (1.45, 2.42)	1×10^{-6}
4 to 6	2921 (22%)	207 (18%)	2.64 (2.03, 3.43)	3×10^{-13}
7 to 9	1824 (14%)	194 (17%)	4.36 (3.33, 5.71)	8×10^{-27}
10 to 12	1003 (8%)	149 (13%)	6.1 (4.6, 8.1)	2×10^{-35}
¿12	1089 (8%)	285 (25%)	11.6 (8.9, 15.1)	2×10^{-73}
**Age 75 years and over (20625 controls, 1217 cases)**				
0	826 (4%)	23 (2%)		
1 to 3	2957 (14%)	67 (6%)	0.77 (0.47, 1.26)	0.3
4 to 6	5014 (24%)	179 (15%)	1.23 (0.78, 1.94)	0.4
7 to 9	4948 (24%)	277 (23%)	1.92 (1.23, 3.01)	0.004
10 to 12	3473 (17%)	259 (21%)	2.65 (1.69, 4.17)	2×10^{-5}
¿12	3407 (17%)	412 (34%)	4.43 (2.84, 6.91)	6×10^{-11}

Table S3. Univariate conditional odds ratios for dispensing of BNF subparagraph codes associated with care home status in controls aged 75 years, filtered to retain rows with at least 50 exposed care home residents

	Independent (20625)	Care home resident (2841)	Odds ratio (95% CI)	p-value
101021 Compound Alginates and proprietary indigestion preparations	1296 (6%)	147 (5%)	0.69 (0.56, 0.85)	6×10^{-4}
102000 Antispasmodic and other drugs altering gut motility	771 (4%)	172 (6%)	1.59 (1.28, 1.98)	3×10^{-5}
106010 Bulk-forming laxatives	680 (3%)	54 (2%)	0.47 (0.34, 0.65)	4×10^{-6}
106020 Stimulant laxatives	1579 (8%)	903 (32%)	4.69 (4.13, 5.32)	1×10^{-125}
106040 Osmotic laxatives	3328 (16%)	1481 (52%)	4.89 (4.40, 5.44)	2×10^{-187}
107010 Soothing haemorrhoidal preparations	202 (1%)	75 (3%)	2.85 (1.97, 4.11)	3×10^{-8}
401010 Hypnotics	989 (5%)	275 (10%)	1.83 (1.54, 2.19)	3×10^{-11}
401020 Anxiolytics	806 (4%)	471 (17%)	4.69 (3.97, 5.53)	1×10^{-74}
402010 Antipsychotic drugs	294 (1%)	528 (19%)	18.9 (15.0, 23.6)	3×10^{-143}
403010 Tricyclic and related antidepressant drugs	1780 (9%)	409 (14%)	2.01 (1.74, 2.34)	2×10^{-20}
403030 Selective serotonin re-uptake inhibitors	1525 (7%)	606 (21%)	3.12 (2.72, 3.58)	4×10^{-60}
403040 Other antidepressant drugs	938 (5%)	511 (18%)	4.92 (4.20, 5.77)	4×10^{-86}
407010 Non-opioid analgesics and compound preparations	8618 (42%)	2000 (70%)	2.84 (2.56, 3.15)	2×10^{-86}
407020 Opioid analgesics	2292 (11%)	635 (22%)	2.23 (1.97, 2.54)	3×10^{-35}
408010 Control of epilepsy	1506 (7%)	288 (10%)	1.71 (1.45, 2.01)	1×10^{-10}
409010 Dopaminergic drugs used in parkinsonism	344 (2%)	83 (3%)	1.88 (1.39, 2.55)	5×10^{-5}
411000 Drugs for dementia	761 (4%)	625 (22%)	7.5 (6.4, 8.8)	3×10^{-132}
501012 Penicillinase-resistant penicillins	1111 (5%)	251 (9%)	1.46 (1.22, 1.75)	4×10^{-5}
501013 Broad-spectrum penicillins	2761 (13%)	616 (22%)	1.78 (1.57, 2.01)	8×10^{-20}
501021 Cephalosporins	226 (1%)	65 (2%)	1.88 (1.34, 2.63)	3×10^{-4}
501080 Sulfonamides and trimethoprim	1619 (8%)	536 (19%)	2.29 (2.00, 2.63)	1×10^{-32}
501130 Urinary-tract infections	1180 (6%)	400 (14%)	2.60 (2.22, 3.04)	8×10^{-33}
502030 Polyene antifungals	212 (1%)	80 (3%)	2.85 (2.05, 3.96)	4×10^{-10}
901011 Oral iron	1585 (8%)	358 (13%)	1.50 (1.29, 1.74)	2×10^{-7}
901020 Drugs used in megaloblastic anaemias	2742 (13%)	683 (24%)	1.77 (1.57, 2.00)	7×10^{-21}
902021 Electrolytes and water	42 (0%)	87 (3%)	9.2 (5.5, 15.4)	2×10^{-17}
904020 Enteral nutrition	365 (2%)	291 (10%)	5.9 (4.7, 7.3)	6×10^{-56}
906026 Thiamine hydrochloride (B1)	266 (1%)	76 (3%)	3.57 (2.55, 5.00)	2×10^{-13}
906040 Vitamin D	4509 (22%)	1138 (40%)	1.54 (1.39, 1.71)	7×10^{-16}
1001010 Non-steroidal anti-inflammatory drugs	960 (5%)	50 (2%)	0.53 (0.39, 0.73)	1×10^{-4}
1001040 Gout and cytotoxic induced hyperiuicaemia	1214 (6%)	90 (3%)	0.60 (0.46, 0.77)	8×10^{-5}
1003020 Rubefacients, topical NSAIDS, capsaicin and poultice	3686 (18%)	660 (23%)	1.28 (1.14, 1.43)	4×10^{-5}

Table S4. Univariate associations of severe COVID-19 with drugs in four subparagraphs of BNF chapter 4, in those not resident in a care home and not treated for cancer in last year, filtered to retain rows with at least 20 exposed individuals

	Controls (30820)	Cases (2062)	Rate ratio (95% CI)	p-value
**Antipsychotic drugs**				
Aripiprazole	38 (0%)	12 (1%)	3.19 (1.61, 6.33)	9×10^{-4}
Olanzapine	95 (0%)	18 (1%)	2.68 (1.57, 4.56)	3×10^{-4}
Levomepromazine	27 (0%)	17 (1%)	9.9 (4.7, 20.6)	1×10^{-9}
Risperidone	123 (0%)	40 (2%)	4.90 (3.26, 7.38)	3×10^{-14}
Haloperidol	17 (0%)	9 (0%)	9.9 (3.6, 26.9)	7×10^{-6}
Chlorpromazine hydrochloride	26 (0%)	7 (0%)	3.00 (1.28, 7.03)	0.01
Amisulpride	21 (0%)	4 (0%)	6.0 (1.7, 21.4)	0.005
**Drugs for nausea and vertigo**				
Prochlorperazine	767 (2%)	59 (3%)	1.37 (1.03, 1.82)	0.03
Cinnarizine	150 (0%)	12 (1%)	1.46 (0.78, 2.71)	0.2
Cyclizine	282 (1%)	42 (2%)	2.62 (1.84, 3.74)	1×10^{-7}
Metoclopramide hydrochloride	159 (1%)	24 (1%)	2.60 (1.62, 4.16)	7×10^{-5}
Betahistine dihydrochloride	367 (1%)	17 (1%)	0.69 (0.41, 1.14)	0.1
Domperidone	54 (0%)	9 (0%)	2.35 (1.10, 4.99)	0.03
**Opioids**				
Morphine	453 (1%)	121 (6%)	4.55 (3.60, 5.76)	1×10^{-36}
Codeine phosphate	564 (2%)	82 (4%)	2.68 (2.05, 3.50)	5×10^{-13}
Tramadol hydrochloride	1136 (4%)	118 (6%)	1.74 (1.41, 2.13)	2×10^{-7}
Fentanyl	102 (0%)	14 (1%)	2.17 (1.18, 4.00)	0.01
Buprenorphine	154 (0%)	9 (0%)	1.18 (0.57, 2.45)	0.6
Oxycodone	249 (1%)	55 (3%)	4.27 (3.05, 5.99)	3×10^{-17}
**Gabapentinoids**				
Gabapentin	969 (3%)	115 (6%)	1.92 (1.56, 2.37)	1×10^{-9}
Lamotrigine	143 (0%)	21 (1%)	2.22 (1.37, 3.60)	0.001
**Control of epilepsy**				
Topiramate	34 (0%)	5 (0%)	1.96 (0.74, 5.17)	0.2
Carbamazepine	174 (1%)	14 (1%)	1.20 (0.68, 2.10)	0.5
Clonazepam	45 (0%)	10 (0%)	3.07 (1.49, 6.32)	0.002
Levetiracetam	137 (0%)	24 (1%)	2.62 (1.64, 4.17)	5×10^{-5}
Sodium valproate	104 (0%)	9 (0%)	1.02 (0.51, 2.06)	1
Phenobarbital	20 (0%)	4 (0%)	2.28 (0.70, 7.39)	0.2
Phenytoin	49 (0%)	11 (1%)	3.08 (1.54, 6.17)	0.001
Primidone	29 (0%)	4 (0%)	1.91 (0.64, 5.70)	0.2
Lacosamide	19 (0%)	2 (0%)	1.52 (0.34, 6.79)	0.6

Table S5. Comparison of associations with exposure only in the most recent 120-day time window with exposure only in the previous 120-day time window (baseline category for rate ratios) for drug classes on the Laporte-Healy list with at least 500 cases and controls exposed only in the earlier time window

Exposure category	Controls (36738)	Cases (4251)	Rate ratio (95% CI)	p-value
**Proton pump inhibitors**				
Non-recent only	1039 (3%)	123 (3%)		
Recent only	1329 (4%)	206 (5%)	1.32 (1.04, 1.68)	0.02
**Antihistamines**				
Non-recent only	759 (2%)	104 (2%)		
Recent only	551 (1%)	102 (2%)	1.32 (0.98, 1.78)	0.07
**Anxiolytics**				
Non-recent only	441 (1%)	112 (3%)		
Recent only	453 (1%)	157 (4%)	1.37 (1.03, 1.82)	0.03
**Tricyclic and related antidepressant drugs**				
Non-recent only	442 (1%)	58 (1%)		
Recent only	396 (1%)	52 (1%)	1.02 (0.69, 1.53)	0.9
**Drugs used in nausea and vertigo**				
Non-recent only	634 (2%)	85 (2%)		
Recent only	607 (2%)	106 (2%)	1.29 (0.94, 1.76)	0.1
**Opioid analgesics**				
Non-recent only	694 (2%)	128 (3%)		
Recent only	777 (2%)	215 (5%)	1.53 (1.20, 1.96)	7×10^{-4}
**Antimuscarinic drugs used in parkinsonism**				
Non-recent only	634 (2%)	85 (2%)		
Recent only	607 (2%)	106 (2%)	1.29 (0.94, 1.76)	0.1
**Drugs for urinary frequency enuresis and incontinence**				
Non-recent only	634 (2%)	85 (2%)		
Recent only	607 (2%)	106 (2%)	1.29 (0.94, 1.76)	0.1

Table S6. Associations of severe COVID-19 with dispensed average daily dose of opioids and proton pump inhibitors, with and without adjustment for prespecified covariates. For opioids the prespecified covariates are care home residence, SIMD quintile and any history of neoplasm. For proton pump inhibitors the prespecified covariates are care home residence, SIMD quintile, any diagnosis of ICD-10 codes K20-K31 (diseases of esophagus, stomach and duodenum), non-steroidal anti-inflammatory drugs, anti-platelet agents and anticoagulants.

			Univariate		Adjusted for covariates	
Average dose	Controls (36738)	Cases (4251)	Rate ratio (95% CI)	*p*-value	Rate ratio (95% CI)	*p*-value
**Opioid-containing analgesics (mg morphine equivalent / day)**						
**Ages 0-74 years**						
0	12039 (91%)	1057 (79%)				
1-20	820 (6%)	157 (12%)	2.24 (1.86, 2.69)	1×10^{-17}	1.95 (1.59, 2.38)	1×10^{-10}
21-50	245 (2%)	68 (5%)	3.26 (2.47, 4.31)	8×10^{-17}	3.06 (2.27, 4.11)	2×10^{-13}
¿50	168 (1%)	54 (4%)	3.66 (2.68, 5.01)	4×10^{-16}	2.96 (2.08, 4.20)	1×10^{-9}
**Ages 75+ years**						
0	20232 (86%)	2301 (79%)				
1-20	2286 (10%)	397 (14%)	1.56 (1.38, 1.75)	3×10^{-13}	1.31 (1.14, 1.50)	1×10^{-4}
21-50	602 (3%)	117 (4%)	1.75 (1.42, 2.15)	2×10^{-7}	1.39 (1.09, 1.76)	0.007
¿50	346 (1%)	100 (3%)	2.72 (2.16, 3.44)	4×10^{-17}	2.17 (1.66, 2.84)	2×10^{-8}
**Proton pump inhibitors (defined daily doses / day)**						
**Ages 0-74 years**						
0	9298 (70%)	721 (54%)				
0.5	1318 (10%)	179 (13%)	1.79 (1.51, 2.14)	6×10^{-11}	1.76 (1.45, 2.13)	7×10^{-9}
1	1513 (11%)	236 (18%)	2.10 (1.79, 2.46)	1×10^{-19}	1.84 (1.54, 2.21)	2×10^{-11}
1.5	650 (5%)	119 (9%)	2.53 (2.04, 3.14)	3×10^{-17}	2.18 (1.72, 2.78)	2×10^{-10}
2 or more	493 (4%)	81 (6%)	2.20 (1.72, 2.83)	5×10^{-10}	1.99 (1.51, 2.62)	1×10^{-6}
**Ages 75+ years**						
0	13409 (57%)	1548 (53%)				
0.5	2832 (12%)	400 (14%)	1.25 (1.11, 1.41)	2×10^{-4}	1.35 (1.18, 1.56)	2×10^{-5}
1	4118 (18%)	545 (19%)	1.16 (1.04, 1.29)	0.008	1.13 (1.00, 1.28)	0.05
1.5	1884 (8%)	264 (9%)	1.22 (1.06, 1.41)	0.006	1.08 (0.92, 1.28)	0.3
2 or more	1223 (5%)	158 (5%)	1.11 (0.93, 1.32)	0.3	1.26 (1.03, 1.54)	0.03

Table S7. Associations of severe COVID-19 with prescribed drugs in BNF chapter 2 by subparagraph code, restricted to those not resident in a care home and filtered to retain rows with at least 50 exposed individuals. Multivariate rate ratios are based on a joint model with all drug classes in the table as covariates

			Univariate		Multivariate	
	Controls (33803)	Cases (2357)	Rate ratio (95% CI)	p-value	Rate ratio (95% CI)	p-value
Cardiac glycosides	821 (2%)	94 (4%)	1.96 (1.54, 2.48)	3×10^{-8}	1.10 (0.85, 1.43)	0.5
Thiazides and related diuretics	3860 (11%)	176 (7%)	0.71 (0.61, 0.84)	5×10^{-5}	0.86 (0.73, 1.02)	0.08
Loop diuretics	3965 (12%)	558 (24%)	2.97 (2.64, 3.33)	7×10^{-76}	2.60 (2.28, 2.96)	1×10^{-47}
Potassium-sparing diuretics and aldosterone antagonists	704 (2%)	95 (4%)	2.08 (1.65, 2.63)	6×10^{-10}	1.20 (0.94, 1.54)	0.1
Potassium sparing diuretics and compounds	71 (0%)	5 (0%)	1.17 (0.45, 3.00)	0.8	1.02 (0.39, 2.69)	1
Drugs for arrhythmias	171 (1%)	19 (1%)	1.71 (1.04, 2.80)	0.04	1.22 (0.73, 2.03)	0.4
Beta-adrenoceptor blocking drugs	8270 (24%)	682 (29%)	1.39 (1.26, 1.54)	4×10^{-11}	1.06 (0.95, 1.19)	0.3
Centrally-acting antihypertensive drugs	70 (0%)	3 (0%)	0.72 (0.22, 2.32)	0.6	0.69 (0.21, 2.28)	0.5
Alpha-adrenoceptor blocking drugs	1283 (4%)	69 (3%)	0.88 (0.68, 1.13)	0.3	0.79 (0.61, 1.03)	0.08
Angiotensin-converting enzyme inhibitors	8009 (24%)	518 (22%)	0.96 (0.86, 1.07)	0.5	0.78 (0.69, 0.87)	2×10^{-5}
Angiotensin-II receptor antagonists	3790 (11%)	221 (9%)	0.88 (0.76, 1.03)	0.1	0.72 (0.62, 0.85)	7×10^{-5}
Nitrates	2843 (8%)	249 (11%)	1.40 (1.21, 1.62)	6×10^{-6}	0.98 (0.83, 1.16)	0.8
Calcium-channel blockers	8256 (24%)	521 (22%)	0.98 (0.88, 1.09)	0.7	1.04 (0.93, 1.16)	0.5
Other antianginal drugs	693 (2%)	70 (3%)	1.56 (1.20, 2.04)	0.001	1.13 (0.85, 1.50)	0.4
Peripheral vasodilators and related drugs	93 (0%)	8 (0%)	1.47 (0.69, 3.15)	0.3	1.26 (0.58, 2.73)	0.6
Parenteral anticoagulants	151 (0%)	23 (1%)	2.34 (1.47, 3.71)	3×10^{-4}	2.08 (1.29, 3.36)	0.003
Oral anticoagulants	3868 (11%)	407 (17%)	1.87 (1.65, 2.11)	3×10^{-23}	1.49 (1.28, 1.74)	4×10^{-7}
Antiplatelet drugs	8809 (26%)	687 (29%)	1.28 (1.16, 1.42)	1×10^{-6}	1.39 (1.23, 1.58)	2×10^{-7}
Lipid-regulating drugs	14485 (43%)	1032 (44%)	1.17 (1.07, 1.29)	8×10^{-4}	1.00 (0.90, 1.12)	0.9

Table S8. Associations of severe COVID-19 with prescribed drugs in BNF chapter 10 by approved name, restricted to those not resident in a care home and filtered to retain rows with at least 50 exposed individuals. Multivariate rate ratios are based on a joint model that includes all drugs in the table as covariates

			Univariate		Multivariate	
	Controls (33803)	Cases (2357)	Rate ratio (95% CI)	p-value	Rate ratio (95% CI)	p-value
**Non-steroidal anti-inflammatory drugs (excluding topical preparations)**						
Diclofenac	253 (1%)	22 (1%)	1.13 (0.72, 1.76)	0.6	1.05 (0.67, 1.64)	0.8
Celecoxib	82 (0%)	11 (0%)	2.20 (1.13, 4.28)	0.02	2.12 (1.09, 4.14)	0.03
Ibuprofen	635 (2%)	56 (2%)	1.24 (0.93, 1.64)	0.1	1.23 (0.93, 1.64)	0.1
Meloxicam	132 (0%)	4 (0%)	0.42 (0.15, 1.16)	0.1	0.43 (0.16, 1.19)	0.1
Etodolac	71 (0%)	9 (0%)	1.60 (0.78, 3.29)	0.2	1.38 (0.66, 2.86)	0.4
Etoricoxib	78 (0%)	12 (1%)	1.94 (1.04, 3.63)	0.04	1.70 (0.90, 3.21)	0.1
**Rheumatic disease suppressant drugs**						
Hydroxychloroquine sulfate	209 (1%)	24 (1%)	2.12 (1.35, 3.31)	0.001	1.71 (1.05, 2.78)	0.03
Methotrexate	328 (1%)	36 (2%)	1.88 (1.31, 2.70)	6×10^{-4}	1.55 (1.04, 2.30)	0.03
**Gout and hyperuricaemia**						
Allopurinol	1501 (4%)	176 (7%)	1.80 (1.51, 2.14)	3×10^{-11}	1.75 (1.47, 2.09)	5×10^{-10}
Colchicine	288 (1%)	35 (1%)	1.69 (1.16, 2.46)	0.006	1.38 (0.94, 2.03)	0.1
**Skeletal muscle relaxants**						
Baclofen	67 (0%)	28 (1%)	5.3 (3.3, 8.4)	2×10^{-12}	5.2 (3.3, 8.3)	4×10^{-12}
